# Cysteine- rich secretory protein 3 (CRISP3), ERG and PTEN define a molecular subtype of prostate cancer with implication to patients’ prognosis

**DOI:** 10.1186/1756-8722-7-21

**Published:** 2014-03-07

**Authors:** Samir Al Bashir, Mohammed Alshalalfa, Samar A Hegazy, Michael Dolph, Bryan Donnelly, Tarek A Bismar

**Affiliations:** 1 Department of Pathology and Laboratory Medicine, University of Calgary and Calgary Laboratory Services, Calgary, AB, Canada; 2 Departments of Oncology, Biochemistry and Molecular Biology, Calgary, AB, Canada; 3 Southern Alberta Cancer Institute and Tom Baker Cancer Center, Calgary, AB, Canada; 4 The Prostate Cancer Center, Calgary, AB, Canada; 5 Department of Urology, University of Calgary, Calgary, AB, Canada; 6 Department of Pathology and Laboratory Medicine, Jordan University of Science and Technology, Irbid, Jordan

**Keywords:** *CRISP3*, *ERG*, *PTEN*, Biological pathways, Prostate cancer, Poorest outcome, Molecular subtypes

## Abstract

Cysteine- rich secretory protein 3 (CRISP3) prognostic significance in prostate cancer (PCA) has generated mixed result. Herein, we investigated and independently validated CRISP3 expression in relation to *ERG* and *PTEN* genomic aberrations and clinical outcome. CRISP3 protein expression was examined by immunohistochemistry using a cohort of patients with localized PCA (n = 215) and castration resistant PCA (CRPC) (n = 46). The Memorial Sloan Kettering (MSKCC) and Swedish cohorts were used for prognostic validation. Results showed, CRISP3 protein intensity to be significantly associated with neoplastic epithelium, being highest in CRPC vs. benign prostate tissue (p < 0.0001), but was not related to Gleason score (GS). CRISP3 mRNA was significantly associated with higher GS (p = 0.022 in MSKCC, p = 1.1e-4 in Swedish). Significant association between CRISP3 expression and clinical outcome was documented at the mRNA but not the protein expression levels. CRISP3 mRNA expression was related to biochemical recurrence in the MSKCC (p = 0.038) and lethal disease in the Swedish cohort (p = 0.0086) and retained its prognostic value in the subgroup of patients with GS 6 & 7. Furthermore, CRISP3 protein and mRNA expression was significantly associated with positive *ERG* status and with *PTEN* deletions. Functional biology analysis documented phenylalanine metabolism as the most significant pathway governing high CRISP3 and *ERG* expression in this subtype of PCA. In conclusion, the combined status of CRISP3, *ERG* and PTEN define a molecular subtype of PCA with poorest and lethal outcome. Assessing their combined value may be of added value in stratifying patients into different prognostic groups and identify those with poorest clinical outcome.

## Introduction

Prostate cancer (PCA) remains a major cancer in the western countries and ranks as the second leading cause of cancer related deaths among men [1]. Although PCA shows different biological behaviour in its disease progression and clinical course, there is strong correlation between pathological prognostic parameters and clinical outcome. In the past decade, several genomic studies have implemented methods to mark specific genomic alterations associated with disease progression and categorize PCA into molecular subtypes with proposed prognostic and therapeutic subgroups. However, currently, those road maps are still being built and refined to better allow physicians to accurately offer better targeted treatment options for PCA patients.

Cysteine- rich secretory protein 3 (CRISP3) is a member of large family of cysteine- rich secretory proteins that are expressed in vertebrates, insects, plants, fungi, and yeast [2-6]. In humans, CRISP3 mRNA is expressed predominantly in salivary gland, prostate, and pancreas, and at much lower levels in epididymis, thymus, ovary, testis, colon, and lacrimal glands [7,8].

*ERG* gene rearrangement is the most abundant genomic aberrations in prostate cancer that accounts for around 50% of clinically localized disease. Furthermore, several studies have implicated *ERG* gene rearrangements with increased rate of *PTEN* deletion. The presence of both *ERG* and *PTEN* aberration has been suggested to signify a distinct molecular subtype of PCA. However, its prognostic implication remains unclear.

Recent studies have associated high CRISP3 with *ERG*- gene rearrangements and *PTEN* deletions and upregulation of transcript levels of CRISP3 has been previously documented in PCA compared to benign prostate tissue using in situ hybridization [2]. This upregulation was also confirmed by few other studies [7,9]. However, data related to the prognostic significance of CRISP 3 has been mixed with conflicting results [10-14] The aim of the current study was to investigate CRISP3 protein expression in large clinical cohort of localized disease and to assess its expression in relation to *ERG* gene rearrangement and *PTEN* deletions and across various stages of disease progression including castration resistant prostate cancer (CRPC). We also aimed to validate the prognostic value of CRISP3 in conjunction to the molecular subtype of PCA with *ERG* and *PTEN* genomic aberrations and to utilize bioinformatics to delineate possible genetic connections and pathways related to this molecular subtype of prostate cancer.

## Material and methods

### Study population and Tissue microarray construction

We utilized two cohorts in this study. The first cohort consisted of 215 patients treated by retro-pubic radical prostatectomy for localized prostate cancer between 1992–2004 with a mean follow-up of 4.8 years (range 0–15.8). Clinical progression was defined as a post-operative serum PSA elevation of >0.2 ng/ml assessed on two occasions following decrease to non detectable levels. Three tissue microarray (TMA) blocks were created from this cohort using a manual tissue arrayer (Beecher Instruments, Silver Spring, MD). Each block was assembled without prior knowledge of any clinical or pathological staging information. From each case, one to nine cores (average 3.3), 0.6 mm in diameter, were sampled from the paraffin embedded tissue blocks, containing benign, high grade intraepithelial neoplasia (HGPIN) and prostate cancer (PCA). We sampled both Gleason pattern originally present in the radical prostatectomy to reflect the average GS from each case. After construction, 4 μm sections were cut and stained with hematoxylin and eosin on the initial slides to confirm the histological diagnosis and grading of Gleason score. The patients’ demographics of this cohort have been previously described [15,16]. The second cohort consisted of 46 patients with castration resistant prostate cancer assembled onto one TMA block. Tissues from those samples were obtained by transurethral resections of prostate preformed to relieve clinical obstructive symptoms thus representing locally advanced disease. The study was approved by the University of Calgary ethics review board who waived informed patients consent. All Clinical and pathological information of patients were collected under this approval.

### Independent validation of mRNA expression data

Two independent prostate cancer expression data were used to validate the findings from the protein expression study. The first data is the Memorial Sloan Kettering Cancer Center (MSKCC) data [17]. The MSKCC sample data were downloaded from Gene Expression Omnibus (GEO) database series GSE21032. All tissues were collected during Radical prostatectomy surgery, snap frozen in liquid nitrogen, and stored at -80°C. Care was taking to identify regions of tumor free from contaminating stroma. The data has the gene expression of 29 normal samples, 131 primary tumor samples after Radical prostatectomy, 19 metastasis samples and 6 cell lines (DU145, LNCaP, LNCaP104R, LNCaP104S, PC3 and VCaP). Samples had a follow up time till biochemical relapse for up to 120 months and are mainly low risk samples. The second data is the Swedish cohort that was downloaded from GEO series GSE16560 that has expression of 281 localized cancer samples; mainly Gleason score 7, and follow up time of overall survival for up 250 months. In the MSKCC data ERG, PTEN status was determined from expression data using PAM clustering implemented in cluster R package. In the Swedish data, ERG was determined by FISH and PTEN was determined from expression data.

### CRISP3 protein expression by immunohistochemistry (IHC)

4 μm thick TMA sections from formalin-fixed paraffin tissue were cut and stained with Leica auto-stainer Bond-Max. The slides were pretreated for deparaffinization followed by heat induced antigen retrieval by Epitope retrieval solution 1 (Leica Microsystems, Buffalo Grove, IL, USA) for 20 minutes, and then incubated for 15 minutes at room temperature with CRISP3 rabbit polyclonal antibody (ProteinTech Group, Inc., Chicago, IL, USA) at 1:100 dilution. Bond polymer Refine Detection kit (Leica Microsystems, Buffalo Grove, IL, USA) was used for DABi detection and counter stain. A multi-tissue control TMA slide containing samples of various tissue organs was previously stained and utilized as positive and negative control to confirm antibody specificity.

### Pathological analysis

All TMA cores were assigned a diagnosis (i.e. benign, HGPIN or PCA) and confirmed independently by three pathologists (SAB, SAH and TAB). Gleason scoring was assessed according to the 2005 ISUP criteria [18]. For each patient, the two predominant patterns were sampled and included on the TMAs blocks for analysis. CRISP3 protein expression was examined by two study pathologists (SAB, SAH) and the intensity of staining was categorized using 4-tiered system according to the following parameters: The staining intensity (0, 1+, 2+, and 3+) for negative, weak, moderate and high intensity plus the fraction of positive tumor cells were documented for each tissue spot. A final score was achieved from these two parameters as follows: Negative(0): absence of CRISP3 staining in 100% of cells; weak (1): intensity of 1+ in > 70% of tumor cells or staining intensity of 2+ in ≤30% of tumor cells; moderate(2): intensity of 1+ in >70% of tumor cells, or staining intensity of 2+ in >30% but ≤ 70% of tumor cells or staining intensity of 3+ ≤30% of tumor cells; strong(3): intensity of 2+ in >70% of tumor cells or staining intensity of 3+ in >30% of tumor cells. As CRISP3 was noticed to be wealy expressed in adjacent stromal cells, when we assessed CRIPS3 expression in our study, we used the expression of adjacent stromal cells as a control for measuring the relative expression of epithelial cells.

*ERG* expression was previously evaluated on this cohort based on positive vs. negative expression as validated and correlated to *ERG* gene rearrangements using the break apart FISH probes (data not shown). *PTEN* deletions were previously evaluated and recorded as not deleted, hemizygous or homozygous deletions by counting 100 nuclei in each core as previously described using a four color FISH probe [19].

### Statistical analysis

Statistical analysis was done to assess various clinical and pathological variables, means and ranges for continuous variables. Chi-square tests were conducted to test for associations between CRISP3 protein expression and Gleason score, surgical margin and pathological stage, *ERG* expression and *PTEN* genomic deletions. Wilcoxon statistical test was used to assess the significance of the difference of CRISP3 mRNA expression across Gleason score, ERG and PTEN status in the MSKCC and Swedish cohorts. CRISP3 protein expression in clinically localized prostate cancer cases association to disease free survival time was assessed using Cox Proportional Hazards regression analysis and the Kaplan-Meier curves along with the log-rank test. In all statistical tests a p value <0.05 was considered significant.

## Results

### Expression of CRISP3 protein in benign, HGPIN, localized and castration resistant prostate cancer

**Figure 1:**
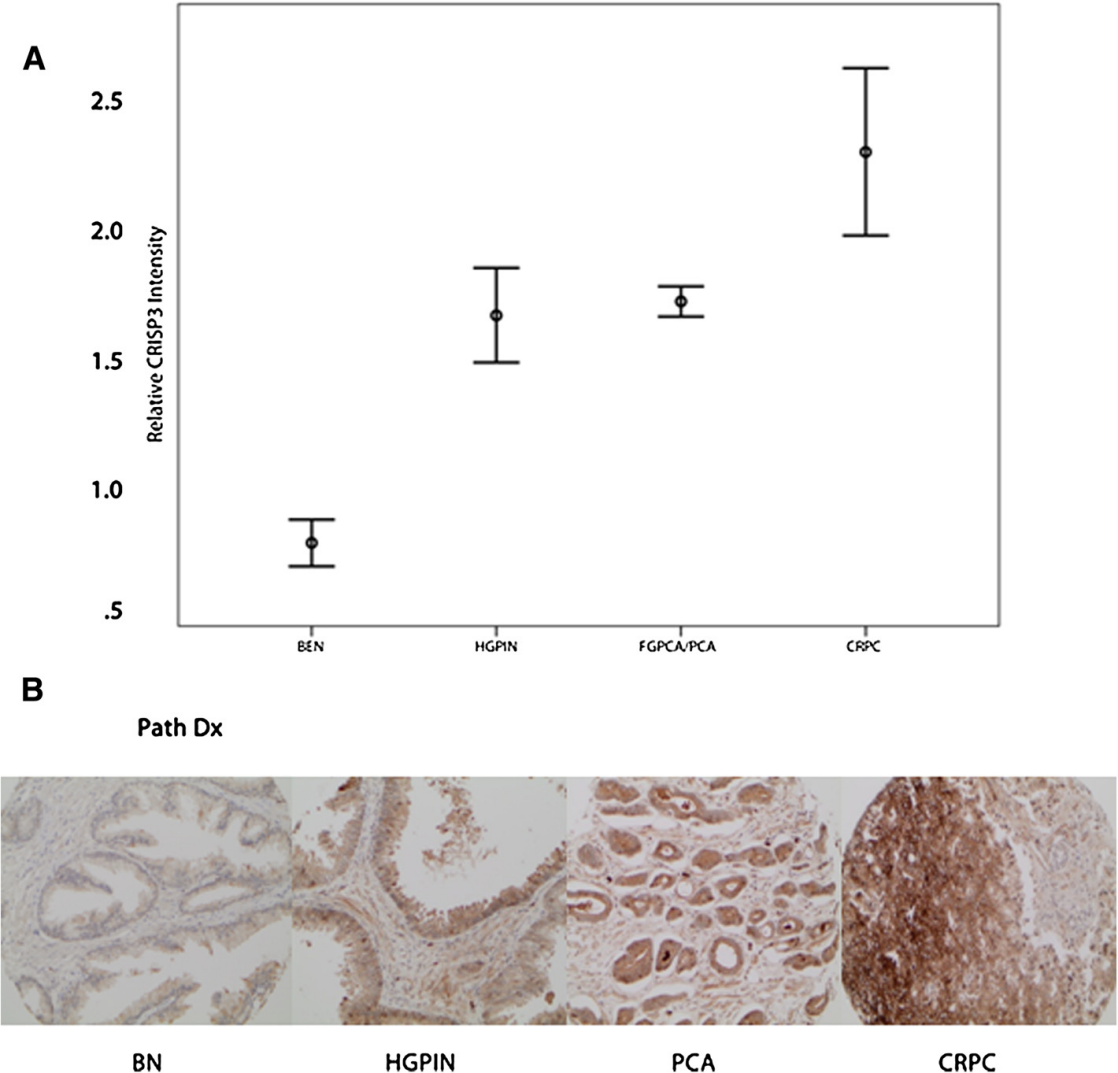
**CRISP3 protein expression across different stages of PCA progression. A**. Error bars of mean intensity of CRISP3 protein expression in benign prostate tissue (BN), high grade intraepithelial neoplasia (HGPIN) and localized prostate cancer (PCA). **B**. Examples of CRISP3 expression in Benign, HGPIN, localized prostate cancer (PC) and castration resistant prostate cancer (CRPC).

A total of 1194 core samples were available for evaluation in the localized cohort; [benign (n = 175), HGPIN (n = 77), PCA (n = 942)]. CRISP3 protein expression (reported as mean ± SD) showed significant increase in PCA (1.71 ± 0.63), compared to benign prostate tissue (1.04 ± 0.42) (p < 0.0001). In HGPIN, mean CRISP3 protein expression was comparable to PCA (1.65 ± 0.56) (p = 0.84) and still significantly higher than benign prostate tissue (P < 0.0001). In the advanced/castration resistant prostate cancer samples (n = 92), CRISP3 protein mean expression levels were the highest with mean intensity of (2.09 ± 0.75) compared to all other types of neoplastic and benign epithelium (p < 0.0001) (Figure 1).

### CRISP3 protein expression in association to Gleason score, surgical margin, pathological stage and PSA biochemical relapse

To investigate the association of CRISP3 protein expression in relation to Gleason score, we grouped samples based on Gleason score sum of individual TMA cores. To enable better correlation of CRISP3 protein expression with GS, CRISP3 protein expression was assessed as binary value, grouping moderate/high expression vs. negative/weak. In this cohort, we did not observe association between CRISP3 protein expression and GS. Moderate/high CRISP3 protein expression was noted in 25/68 (36%) of GS < 7 compared with 52/145 (35.8%) of GS ≥ 7.

**Figure 2:**
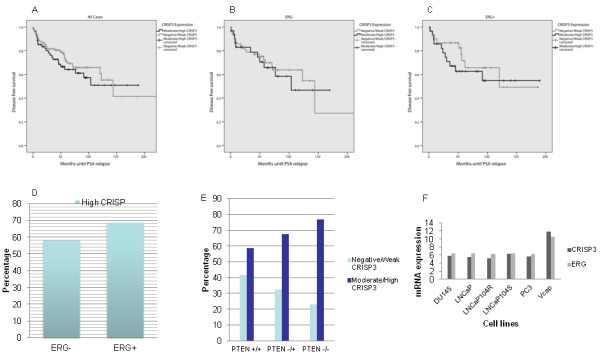
**Kaplan Meier curves of CRISP3 protein. A**. Disease free survival for CRISP3 protein expression in all cases. **B**. Disease free survival for CRISP3 protein expression in *ERG* negative cases, **C**. Disease free survival for CRISP3 in *ERG* positive cases. **D**. Error bars showing percent cases with CRISP3 protein expression related to *ERG* rearrangements in prostate cancer. **E**. Error bars showing percent of CRISP3 expression in *PTEN* intact (PTEN+/+), hemizygous (PTEN+/-) and homozygous (PTEN-/-) *PTEN* deletions prostate cancer. **F**. mRNA expression levels of CRISP3 and *ERG* in multiple cell lines.

Similarly, we did not observe significant relation to any other clinical or pathological parameters. However, a non-significant trend toward inverse relation between moderate/high CRISP3 protein expression and higher pathological stage was noted. Herein, 19/68 (27%) moderate/high CRISP3 protein expression were noted in pT3 vs. 54/135 (40%) in pT2 (p = 0.09). CRISP3 protein was not related to disease free survival as measured by post serum PSA elevation of ≥ 0.2 ng/ml (Figure 2). Table 1 illustrates patients’ demographics in relation to CRISP3 protein expression.

**Table 1:** Patients demographics of the localized cohort stratified based on CRISP3 expression

***Parameter***	***Negative/weak (n = 137)***	***Moderate/high (n = 78)***	***p-value***
*Age (years) (mean; range)*	64.53 (43–81)	63.58 (47–78)	0.280
*Pre-PSA level (ng/ml)*			0.212
*= < 10*	43 (68%)	28 (80%)	
*>10*	20 (32%)	7 (20%)	
*Gleason summary*			0.750
*<7*	43 (32%)	25 (33%)	
*3 + 4*	43 (32%)	24 (31%)	
*4 + 3*	31 (22%)	20 (26%)	
*>7*	19 (14%)	8 (10%)	
*pT-stage*			0.091
*pT2*	81 (62%)	54 (74%)	
*pT3*	49 (38%)	19 (26%)	
*Surgical margin*			0.285
*Negative*	69 (53%)	45 (61%)	
*Positive*	61 (47%)	29 (39%)	

### CRISP3 protein expression in relation to ERG expression and PTEN genomic deletions

**Figure 3:**
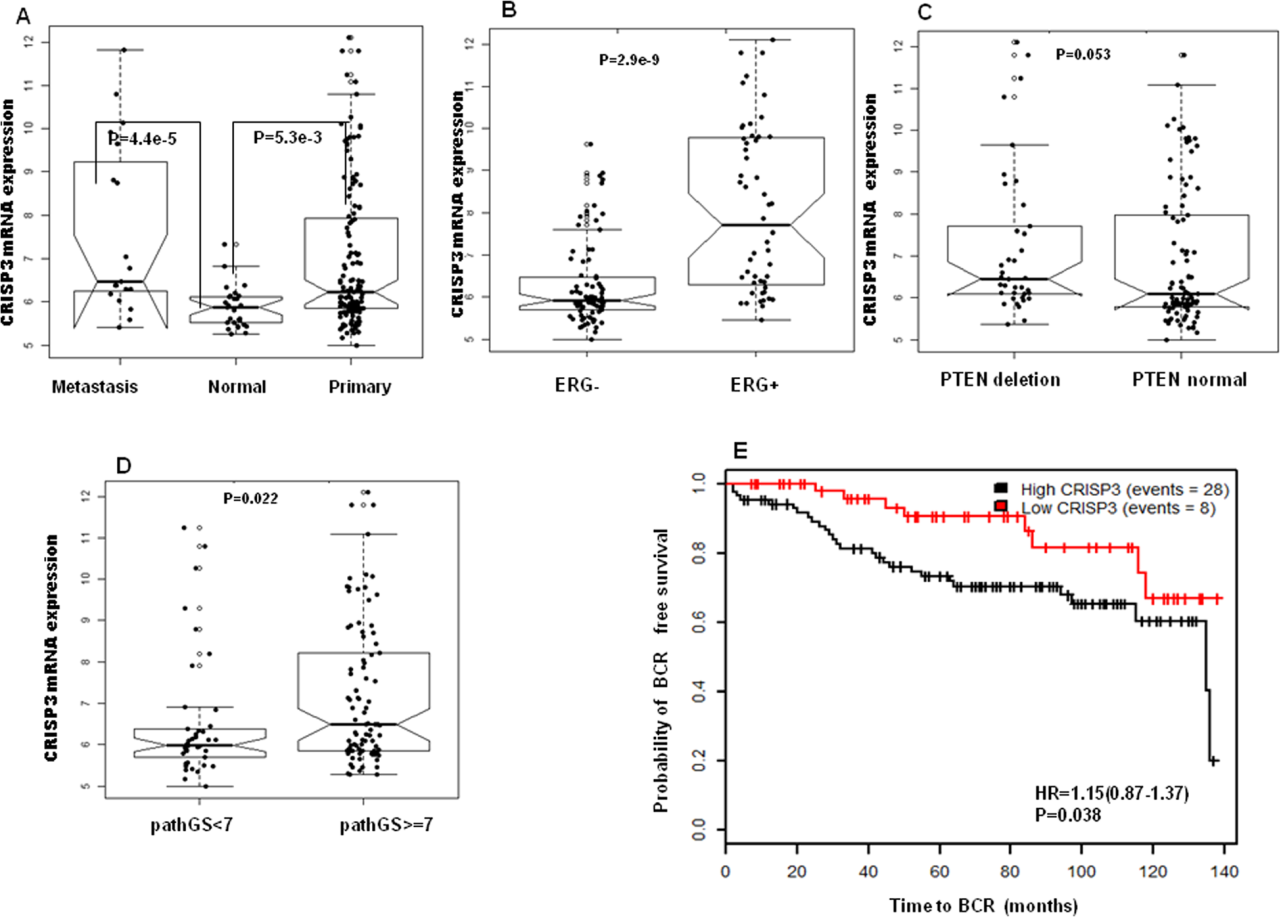
**Expression of CRISP3 mRNA in MSKCC data. A**. Association of CRISP3 to prostate cancer progression, **B**.CRISP3 association to *ERG*, **C**. CRISP3 association to PTEN. **D**. Association of CRISP3 to Gleason score. **E**. Kaplan Meier of CRISP3 mRNA expression to time till biochemical relapse in MSKCC Cohort.

To assess the association between CRISP3 protein and *ERG* expression, 812 cores of localized prostate cancer were assessed for the dual expression of both markers. There was significant association between higher CRISP3 protein expression and *ERG* expression. Moderate/high CRISP3 protein expression was present in 217/316 (69%) in *ERG* positive vs. 287/496 (58%) in *ERG* negative tumor cores (p = 0.002) (Figure 3A).

Since it was previously documented that *ERG* genomic rearrangements are enriched for *PTEN* deletions [20], we sought to investigate the association of CRISP3 with *PTEN* genomic deletions based on the type of *PTEN* deletion present within the individual core sample. Overall, there was significant association between CRISP3 protein expression and *PTEN* deletions (71% vs. 58%) (p = 0.01). To further investigate the relation of the type of *PTEN* genomic deletions and CRISP3 protein expression, we grouped the samples into *PTEN* intact, hemizygous *PTEN* deletions and homozygous *PTEN* deletions. In this cohort, higher CRISP3 protein expression was significantly and positively associated with the degree of *PTEN* genomic aberrations (not deleted vs. hemizygous vs. homozygous deletions) (Table 2). Moderate/high CRISP3 protein expression was present in 279/477 (58%) *PTEN* intact tumors vs. 92/136 (68%) *PTEN* hemizgous deletions vs. 40/52 (77%) in *PTEN* homozygous deleted PCA (P = 0.01) (Figure 3B).

**Table 2:** **Association between CRISP3 immunostaining and PTEN intact, hemizygous *PTEN *** **deletions and homozygous *PTEN *** **deletions**

	***Negative/weak CRISP 3 expression n (%)***	***Moderate/high CRISP 3 expression n (%)***	***Total (n)***	***p-value***
*PTEN not deleted (n)*	304(50%)	310(50%)	614	<0.0001
*PTEN hemizygous deletion ( n)*	53(35%)	97(65%)	150	
*PTEN homozygous deletion (n)*	13(24%)	42(76%)	55	
*Total (n)*	370(45%)	449(55%)	819	

Cases assessed as individual TMA cores.

### CRISP3 expression in PCA Cell lines

To validate CRISP3 mRNA expression relative to *ERG* status, we investigated the mRNA expression of CRISP3 and *ERG* in multiple cell lines from the MSKCC data (GSE21032). Both CRISP3 and *ERG* showed two-fold increase in VCaP cell line which harbors *TMPRSS2-ERG* rearrangement (Figure 3C). Using VCaP cell line to validate whether CRISP3 mRNA expression is a direct target of the *ERG* transcription factor , Ribeiro et al. detected three putative ETS-binding-sites containing-regions of the CRISP3 promoter in the *ERG*-bound chromatin, supporting direct link between *ERG* and CRISP3 [13]*.*

### CRISP3 expression in independent gene expression data of the MSKCC and Swedish prostate cancer cohorts

**Figure 4:**
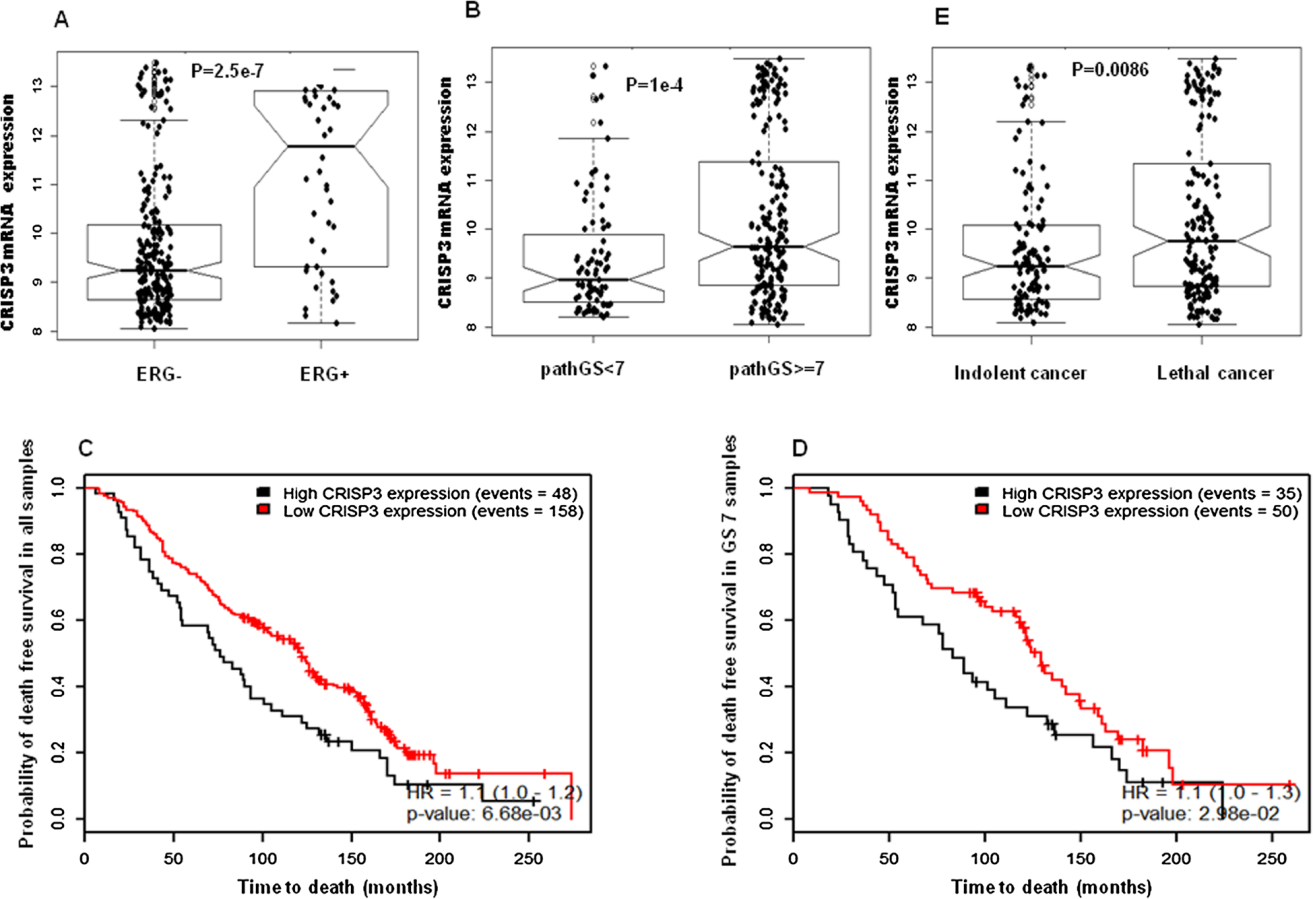
**Expression of CRISP3 mRNA in Swedish data. A**. Association of CRISP3 to *ERG*, **B**. Association of CRISP3 to Gleason score, **C**. Kaplan Meier demonstrating association of CRISP3 to time to death in all samples regardless of GS, **D**. Kaplan Meier association of CRISP3 to time to death in GS = 7 subgroup. **E**. Association of CRISP3 mRNA expression to cancer lethality in the Swedish cohort.

We next characterized the expression of CRISP3 with prostate cancer subtypes and clinical variables at the mRNA level. We used two public mRNA gene expression data MSKCC (GSE21032) and the Swedish cohort (GSE16560) and used Wilcox test to assess the statistical significance of the association between CRISP3 and other clinical variables. Analyzing the mRNA expression of CRISP3 in normal and PCA showed that CRISP3 mRNA expression is significantly upregulated in PCA (p = 0.0053) and metastasis (p = 4.4e-5) (Figure 3A). CRISP3 mRNA expression was also significantly associated with *ERG* status as determined by mRNA ERG expression (p = 2.9e^-9^) and *PTEN* deletions as determined by mRNA PTEN expression (p = 0.053) (Figure 3B,C) in the MSKCC data, and significantly associated with *ERG* gene rearrangements in Swedish cohort determined by FISH (p = 2.5e-7) (Figure 4A).

Investigating relation to Gleason score and grouping cases into <7 vs. ≥ 7, high CRISP3 mRNA expression was associated with high GS in both MSKCC (p = 0.022) (Figure 3D) and the Swedish (p = 1e^-4^) (Figure 4B). High CRISP3 mRNA expression, determined by PAM clustering, also was associated with time to biochemical recurrence (BCR) after Radical prostatectomy (p = 0.038, HR: 1.15) (Figure 3E), but not BCR event (data not shown). However, when investigating association to lethal disease, high CRISP3 mRNA expression was associated with time to death (p = 0.0066) (Figure 4C) in all samples of the Swedish cohort and the subsamples in Gleason score 7 (p = 0.029) (Figure 4D). Additional analysis supported the implications of high CRISP3 mRNA expression in prostate cancer lethality (p = 0.0086) (Figure 4E).

### The biological pathways of genes deregulated in PCA subset with ERG positive and high CRISP3 mRNA expression

**Figure 5:**
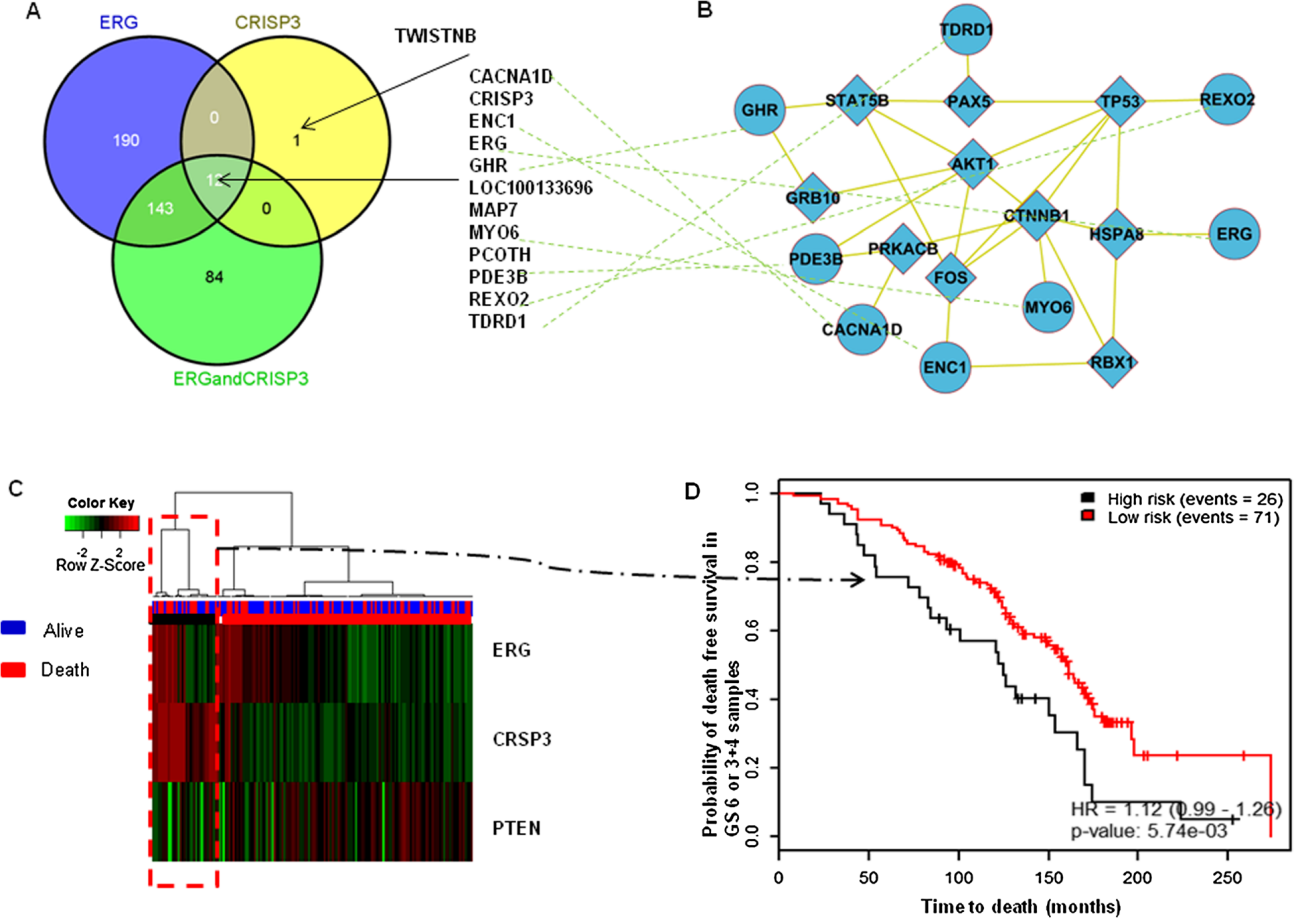
**Cross-talk between CRISP3 and *ERG*.** 12 genes are implicated in both CRISP3 and *ERG* samples **(A)**. The 12 genes are connected to p53, CTNNB1, AKT1 and other cell proliferation genes **(B)**. A heatmap showing the prognostic value of combining the status of *ERG*, PTEN and CRISP3 in samples with GS6 or 3 + 4 in the Swedish data. Hierarchical clustering showed that the three genes define distinct subgroups of patients with GS6 or 3 + 4 **(C)**. The cluster that is enriched with high *ERG*, CRISP3 and low PTEN showed poorest survival.

To analyze the cross-talk between *ERG* and CRISP3, we identified the differentially expressed genes from MSKCC data between *ERG* positive vs. *ERG* negative, high CRISP3 mRNA expression vs. low CRISP3 mRNA expression and *ERG* positive/ high CRISP3 mRNA expression vs. others (Figure 5A). To select differentially expressed genes, we used student *t*-test, adjusting for multiple corrections using bonferroni method. Herein, we identified a group of 12 genes that are implicated in both CRISP3 and *ERG* subgroups. These 12 genes are highly correlated and interacting with AKT1, TP53, CTNNB1 and FOS network of genes extracted from the FI reactome functional protein networks implemented in Cytoscape (Figure 5B) suggesting a critical role of CRISP3 in cell proliferation [21].

Expression of *ERG* and *CRISP3* mRNA expression is significantly correlated. Low CRISP3 mRNA expression was associated with ERG negative, while high CRISP3 mRNA expression was associated with *ERG* positive. However, 50% of *ERG* positive samples has low CRISP3 mRNA expression, suggesting that CRISP3 expression is conditionally dependent on the expression of *ERG* or CRISP3 overexpression is a consequence event of ERG rearrangement.

We further investigated the functional biology of the 84 genes that are implicated in the *ERG* positive –high CRISP3 PCA subtype. These 84 genes were differentially expressed in only samples with high CRISP3 mRNA expression and ERG positive samples, and define a distinct molecular profile of samples. SPINK1 is found to be differentially expressed in the ERG-positive-high CRISP3 PCA subtype. Using Gene set enrichment analysis [22], the 84 genes were enriched in ribosomal proteins (p = 4.1e-4) and translational elongation process (p = 0.018) (Table 3). Using network-based enrichment analysis tool (http://www.enrichnet.org/) to characterize the biological pathways governing these genes, phenylalanine metabolism was the most significant pathway that governs the 84 genes. This depicts the molecular mechanism underlying the combination of ERG and CRISP3 regulation.

**Table 3:** **Top deregulated genes in high CRISP3 expression, *ERG *** **positive samples and the pathways they are involved in**

**Pathway**	**Top genes in the 84 set**
mTORC mediated signaling	EIF4B
Unwinding of DNA	GINS2
Steroid hormone biosynthesis	HSD17B3
Peptide chain elongation	RPL4, RPL5, RPS3, RPL17
Protein translation and ribosomes	EIF4B, RPL4, RPL5, RPS3, RPL17
Deadenylation of mRNA	EIF4B
Signaling by BMP	BMPR1B

### Potential clinical utility of combining ERG, CRISP3 and PTEN

To confirm the significant association of CRISP3, *ERG* and PTEN, we assessed the prognostic value of combining *ERG*, PTEN and CRISP3 expression. The mRNA expression levels of the three genes were extracted from the Swedish cohort and hierarchical clustering was used to group all samples based on the three genes’ expression. Clustering confirmed that samples enriched with high *ERG*, *high CRISP3* and low *PTEN* have marginally poor outcome (p = 0.053). We then focused on patients with low risk tumors; with Gleason score 6 or (3 + 4) to investigate whether this signature is able to stratify patients within this prognostic ambiguous group (Figure 5C), patients with the signature of high *ERG*, high *CRISP3* and low *PTEN* were significantly associated with poorest clinical outcome (p = 0.0057) (Figure 5D).

## Discussion

CRISP3 belongs to a large family of cysteine –rich secretory proteins which are poorly characterized extracellular proteins [14]. This family is highly expressed in salivary glands and in the male reproductive tract, most of which are under strong androgen-dependency [13]. In humans, CRISP3 was first described in neutrophils, but later it was shown to be highly expressed in several exocrine glands, including salivary glands, pancreas and prostate [2-6] and within different cells of the immune system including pre-B cells, neutrophils, and eosinophils [23,24].

CRISP3 has been previously reported to be overexpressed in prostate cancer [7,9,25]. Using in situ hybridization, Kosari et al. al reported that CRISP3 mRNA expression is epithelial-specific and is up-regulated in prostate cancer compared with benign prostate tissue [2]. Ernst et al. found that majority of *CRISP3* production came from the prostatic epithelium, rather than the surrounding stromal tissue [9]. Others noted that CRISP3 expression increases more than 50 fold in prostate cancer [7]. In our immunohistochemistry study we document that CRISP3 protein levels are highly expressed in all neoplastic prostate epithelium compared with benign prostate tissue. We also report for the first time that CRISP3 protein expression is highest in the CRPC category. This suggests a key role of CRISP3 in AR-independent mediated transcriptional program.

The association between CRISP3 and clinico-pathological parameters has been investigated in several previous publications, but with mixed results. Table 4. Summarizes the results of CRISP3 protein expression in correlation with clinico-pathological parameters from five previous studies in addition to our own [10-14]. Bjartell et al. [26] found increased CRISP3 protein expression in higher grade PCA (Gleason 4 and 5). However in a subsequent study by the same author, no significant difference in GS between patients with positive and negative CRISP3 was noted [12]. The association between GS and CRISP3 protein expression was only evident in one more study by Grupp et al., but was not demonstrated in three other. In our study, we were not able to document any association between CRISP3 and GS at the protein level; however, at the mRNA levels we document significant positive association with higher GS. One possible explanation to this discripancy could be related to the inclusion of adjacent stromal cells in the mRNA expression assessment (Taylor et al.) where stromal cells had indeed expressed lower levels of CRISP3, though the authors dissected the samples carefully to ensure minimal contamination of tumor samples with non-neoplastic tissue. Moreover, the relation of CRISP3 to other pathological parameters, was only documented in one study (pathological stage and surgical margin) with all other 4 studies including ours failing to show such association. These data suggest either that morphological assessment of CRISP3 protein expression is inaccurate due to potential heterogeneity or protein abundance or that CRISP3 is not actually related to GS and other pathological parameters and hence provide added value beyond those of current pathological parameters. The latter is more likely given that CRISP 3 mRNA expression was significantly associated to time of biochemical relapse and lethal disease in the MSKCC and Swedish cohorts.

**Table 4:** Results of CRISP3 expression in correlation with clinico-pathological parameters from comparatives studies

***Study***	***Correlation of CRISP 3 expression with clinico-pathological parameters***			
	***Gleason score***	***Pathological stage***	***Surgical margins***	***Outcome***
Ribeiro et al. [13]	No	No †	Not Tested	N/A
Grupp et al. [14]	Yes	Yes	Yes	Univariate
Dahlman et al. [10]	No?	No?	No?	Trends*
Hoogland et al. [11]	No	No	No	N/A
Bjartell et al. [12]	No	No	No	Yes^¶^
Al-Bashir et al.	No	No	No	No^μ^

*There was a trend that patients with high CRISP3 expression had higher risk of biochemical recurrence.

^¶^Adding CRISP3 expression to current parameters does not provide any added significance to the prediction.

†Using non-parametric tests on the qRT-PCR data from the validation series.

^μ^Based on protein expression. However, mRNA CRISP3 levels were prognostic based on the validation within Taylor and Swedish cohorts.

CRISP3 has been reported to be one of the top genes that are linked to *TMPRSS2-ERG* positive prostate cancer with about 53-fold increase when compared with fusion negative prostate cancer [13]. Our study confirms this strong association.

It is well known that up to 40% of human prostate cancers show loss of at least one copy of the *PTEN* gene via submicroscopic deletion [27-29] and that the percent of tumors harboring *PTEN* deletions increases with disease progression and in advanced castration resistant disease [19,20]. Moreover, the complexity of *PTEN* deletions increases with higher Gleason score and is tightly associated with *ERG* rearrangement [8,20,28-31]. In this study, we carried out a detailed investigation between CRISP3 protein expression and the type of *PTEN* deletions which was only partially addressed in the study by Grupp et al. [14], where they documented significant association between *PTEN* deletions and CRIPS3 protein expression. However, the relation between the type of *PTEN* deletion and CRISP3 protein expression was not addressed. Herein, we confirm significant increase in CRISP3 protein expression not only in *PTEN* deleted tumors, but also document that this expression is also connected and increases with the degree and severity of *PTEN* deletion (hemizygous vs. homozygous) (65% vs. 76%) respectively.

We also investigated the potential linkage between CRISP3 mRNA expression and *ERG* gene rearrangements and identified a 12-gene signature that is implicated in both *CRISP3* and *ERG* subgroup of PCA. We also investigated the functional biology of an 84 genes that are implicated in the *ERG* positive –high *CRISP3* mRNA expression PCA subtype, documenting phenylalanine metabolism as the most significant and likely pathway that governs these genes. This correlation suggests that *CRISP3* is a major target of *ERG* that is strongly overexpressed in PCA.

Although our study did not provide any prognostic implication of CRISP3 expression in prostate cancer at the protein level, we were able to confirm significant prognostic value for CRISP3, in the MSKCC and Swedish cohorts. This may be due to heterogeneity or the methods used to assess for protein expression and highlight the need for more accurate measurement of protein expression, likely through the use of image analysis systems. The tight link between CRISP3 expression, *ERG* gene rearrangements and/ or *PTEN* deletions in the three cohorts of prostate cancer studied, suggest the presence of distinct molecular alterations in this subgroup of tumors that may have additional clinical implication if assessed collectively.

In this study, we validated the prognostic value of the combined status of the three genes by clustering the samples in the Swedish data into multiple clusters. The clustering showed that the status of the three genes can stratify patients into distinct subgroups with distinct outcome. The cluster that is enriched with high *ERG*, high CRISP3 and low PTEN demonstrated the poorest survival outcome and this was evident in the group of patients with GS6 & (3 + 4), which is the most group likely to benefit of signature biomarker. This finding is novel and has not been shown in any previous study, including the study by Grupp et al. in which association of CRISP3 was noted with ERG and PTEN, but it failed to document any added prognostic value for the three markers combined [14].

In Summary, in this study we confirmed significant association between CRISP3 expression and prostate cancer progression as well as a strong link between CRISP3 expression and each of *ERG* expression and *PTEN* genomic deletions. In addition, we documented and detailed potential genes that are tightly connecting this molecular subtype of PCA. More importantly, we confirm and for the first time, that tumors defined by CRISP3, *ERG* and PTEN signify a molecular subtype of PCA with the poorest clinical outcome. This suggests that the combination of the three genes could be used as a molecular test to stratify patients’ risk. Further studies to elucidate the molecular network connecting CRISP3, *ERG* and *PTEN* and to validate their combined prognostic role may be of added value in stratifying patients with prostate cancer, specifically those with Gleason score 6&7.

## Competing interests

M. A is employer of GenomeDx Bioscience Inc. The other authors declare that they have no competing interests.

## Authors’ contributions

SA carried out immunostaining, pathological analysis and wrote initial manuscript. MA performed the bioinformatics analysis. SH carried out pathological analysis. MD performed statistical and survival analysis. BD designed the study. TB designed the study, carried out pathological analysis and wrote the initial manuscript. All authors read and approved the final manuscript.
